# Associations among Fall Risk Appraisal, Body Composition, and Physical Activity in Older Adults

**DOI:** 10.1093/geroni/igab046.3565

**Published:** 2021-12-17

**Authors:** Ladda Thiamwong, Rui Xie, Renoa Choudhury, Joon-Hyuk Park, Oscar Garcia, Aleatha Rossler, Jeffrey Stout

**Affiliations:** 1 College of Nursing, University of Central Florida, Orlando, Florida, United States; 2 University of Central Florida, Orlando, Florida, United States

## Abstract

One-third of older adults have maladaptive fall risk appraisal (FRA), a condition in which there is a discrepancy between perceived fall risk or levels of fear of falling (FOF) and physiological fall risk (balance performance). We aimed to examine the associations among FRA, body composition, and physical activity (PA) using Assistive Health Technology, including the Bioelectrical Impedance Analysis, BTrackS Balance System, and activity monitoring devices. We evaluated 124 older adults with a mean age of 74.81 (SD=7.31, range 60 to 96), 77% were female, and 72% had no history of falls. The multinomial logistic regression was used to analyze the data. FRA was classified into 4 quadrants, and we found 47% of rational FRA (low FOF and normal balance), 19% of incongruent FRA (low FOF despite poor balance), 18% of irrational FRA (high FOF despite normal balance), and 16% congruent FRA (high FOF and poor balance). We found these following variables are associated with FRA: accelerometer-based moderate to vigorous physical activity (MVPA: mins), self-reported PA score (strength & flexibility), had difficulty walking up 10 steps without resting (resistance), had difficulty walking several city blocks (ambulation), left-hand average handgrip strength, CDC fall risk score, Senior Technology Acceptance (STA) score and body composition including Body Fat Mass (BFM), Percent Body Fat (PBF), Body Mass Index (BMI), Whole Body Phase Angle, Skeletal Muscle Mass (SMM) and Skeletal Muscle Index (SMI). Our results support the efficacy of using Assistive Health Technology on screening individuals with maladaptive FRA with targeted interventions to reduce fall risk.

